# “What say ye gout experts?” a content analysis of questions about gout posted on the social news website Reddit

**DOI:** 10.1186/s12891-017-1856-y

**Published:** 2017-11-23

**Authors:** Christina Derksen, Anna Serlachius, Keith J. Petrie, Nicola Dalbeth

**Affiliations:** 1 Department of Psychological Medicine, University of Auckland, Auckland, New Zealand; 2 Department of Medicine, Faculty of Medical and Health Sciences, University of Auckland, 85 Park Rd, Grafton, Auckland, New Zealand

**Keywords:** Social media, Gout, Education

## Abstract

**Background:**

Social media is increasingly used by patients to source information for managing chronic disease. The aim of this study was to understand patient information needs about gout by a content analysis of questions posted on the social news website Reddit.

**Methods:**

We analysed questions posted onto the ‘Gout sufferers unite’ subreddit site. Two reviewers coded questions into categories (inter-reviewer kappa 0.70), with discordant coding resolved by a third reviewer. Data were analysed by calculating the frequency of questions within the categories. Where relevant, categories were further separated into sub-categories to allow organisation and interpretation of the data.

**Results:**

We analysed 359 questions in 287 posts by 213 individuals. A wide range of questions arose. The single most common category related to uncertainty of diagnosis (22.3% questions), with questions about disease management common. Information-seeking about medications was generally cautious, with questions about side-effects, risk of flares after starting urate-lowering therapy, and decision to start urate-lowering therapy. Community users experiencing flares posted questions about flare management, including medications, sometimes in real-time. Dietary management questions included the effectiveness of dietary changes as a management strategy, choice of alcoholic beverage, and weight loss strategies. Questions about serum urate levels were rare (2.8% questions).

**Conclusions:**

Questions about gout posted on the subreddit site most often related to uncertainty about symptoms and disease management strategies, with infrequent questions about serum urate testing, results or targets. These findings may inform development of strategies to address the information needs of people with gout.

## Background

Gout is a chronic disease of monosodium urate crystal deposition that typically presents as recurrent flares of painful inflammatory arthritis [1]. It is the most common form of inflammatory arthritis affecting men, with increasing prevalence worldwide [2]. The central strategy for effective long-term management of gout is urate-lowering therapy, with long-term sub-saturation urate concentrations leading to crystal dissolution and prevention of flares [3].

Gout is poorly controlled worldwide, with low adherence to urate-lowering therapy and infrequent achievement of serum urate target [4]. Strategies to improve gout management may substantially reduce the community impact of this condition. Improving patient understanding about the disease is an important element of gout management [5]. However, both written and online patient resources about gout are lengthy, require a high reading level, and do not address key aspects of disease [6–8]. Furthermore, the content of these resources may not align with patients’ current concerns and questions about disease. There are very little available data on the education needs of people with gout; one small qualitative study reported that patients wanted to know more information about the aetiology of gout, treatment goals, long-term consequences, and lifestyle modification [9].

Social media is widely and increasingly used by patients to source information about disease [10]. As the household use of the internet has increased, there has been an increase in the number of online support groups for patients with chronic illness [11]. Now from their home computer or smartphone, patients can participate in discussions with other patients who have a similar illness. New social media sites like reddit.com have appeared where registered users can submit content and seek advice. In 2016 Reddit had 542 million monthly visitors and was ranked the 25th most visited website in the world with 8 billion page views per month [12]. Within Reddit, areas of different content, such as peer support communities, appear as a subreddit topic.

While it is clear patients are increasingly using social media platforms to inform disease management, very little research has examined the types of questions patients seek answers for on these platforms. The aim of this study was to understand patient information needs about gout by a content analysis of questions posted on Reddit.

## Methods

We analysed questions posted onto the ‘Gout sufferers unite’ subreddit. This open access subreddit has existed for five years, and had 1811 ‘Gout friends’ at the time of writing. We analysed questions in the “Hot” section of the subreddit posted from September 4th 2015 to August 23rd 2016. Posts were extracted from the website with information including title, text, username, and questions in the text. Posts that did not include a question were not extracted for analysis.

A sample of questions were initially reviewed by four reviewers (CD, AS, KP, ND), and 13 categories (themes) were agreed upon. Two reviewers (CD and AS) then coded all questions into the categories. Kappa values were analysed using SPPS v23 (IBM Corp., Armonk, USA). Kappa (SE) for inter-reviewer agreement was 0.70 (0.026). Discordant coding was resolved by review by a third author (ND). Data were analysed by calculating the frequency of questions with the categories. After coding and agreement, categories were reviewed and where relevant, further separated into sub-categories to allow organisation and interpretation of the data.

According to the University of Auckland Human Participants Ethics Committee policy, this work did not require ethical approval, as it involved analysis of publically available data. Consent was not obtained from individuals or Reddit, as the posts are available in the public domain and were made voluntarily from individuals who could not be identified.

## Results

### Posts and categories

We analysed 359 questions in 287 posts by 213 individuals. The majority of individuals (171/213, 80.3%) submitted a single post. The mean number of posts per individual was 1.34 (range 1–8). For most posts, it was not possible to determine disease duration of gout, but a new or very recent diagnosis was mentioned by 41/213 (19.2%) users. A wide range of questions arose. The single most common category was about uncertainty of diagnosis (22.3% questions), with management categories (questions about medication, dietary advice, non-pharmacological management, and non-specific management) also common (Table 1). Questions about financial issues, information sources, and perceptions of gout were less common. Notably, only 10/359 (2.8%) questions were about serum urate levels.

**Table 1:** Categories (% questions)

Category	n (%)
Uncertainty about symptoms	80 (22.3%)
Medications	55 (15.3%)
Diet	44 (12.3%)
Non-pharmacological management	37 (10.3%)
Non-specific management	37 (10.3%)
Triggers	24 (6.7%)
Seeking social support	20 (5.6%)
Financial and employment issues	16 (4.5%)
Miscellaneous	13 (3.6%)
Causes of gout	12 (3.3%)
Serum urate testing	10 (2.8%)
Information sources	7 (1.9%)
Social perception of gout	4 (1.1%)

### Questions related to uncertainty about symptoms

The most common single category was uncertainty about symptoms. Within this category, there were three main sub-categories; uncertainty about the diagnosis of gout, atypical gout symptoms, and the timeline of disease. Examples of questions are shown in Table 2. Some community users described a course of illness different from the ‘classical’ presentation of gout, including lack of redness or swelling, prolonged symptoms, or presentation of arthritis in atypical joints, which raised uncertainty about the diagnosis. Questions about the timeline of disease, including the duration and temporal pattern of flares, were also posted, including questions about the duration of a single flare and the likelihood of having further flares after the first flare.

**Table 2:** Questions related to uncertainty about symptoms

Sub-category	Examples of questions
Uncertainty about the diagnosis of gout	*“Is that normal for gout? Does it stay sore for months? Or should I have it rechecked by a different doctor to see if there’s an injury?”* *“Doc says gout. I’m not sure. - What say ye gout experts?”*
Atypical symptoms	*“Hi all, Is chronic pain in the toe joint a common thing?”* *“Anyone ever had it in your lower back before?”* *“Anyone else have gout flare-ups on the back of their heel?”* *“Gout switching between extremities. - Has anyone else experienced this?”*
Timeline of symptoms	*“What is everyone’s experience with residual pain/discomfort? (…) Can I expect things to slowly clear up as the days progress?”* *“If I had one attack, will I always have them? Does it ramp up?”*

### Questions about medications

Questions about medications was the next most common category. There were 55 (15.3%) questions about medications. These included questions about specific named drugs, most commonly allopurinol (33 questions) and colchicine (15 questions). Questions about medications were generally cautious or negative, with key sub-categories of medication side effects, risk of flares after starting urate-lowering therapy, and deciding whether to start urate-lowering therapy. Community users experiencing flares posted questions about medications for flare treatment in real-time. Examples of questions are shown in Table 3. Additional questions related to specific drug dosing (primarily related to allopurinol and colchicine), selection of specific urate-lowering drugs, when to start urate-lowering therapy, the need for anti-inflammatory prophylaxis therapy, and drug interactions.

**Table 3:** Questions about medications

Main sub-categories	Examples of questions
Medication side effects	*“I’m just curious if anyone has had any bad experiences with allopurinol, or if there’s some side effect that I’m missing.”*
Flares after starting urate-lowering therapy	*“Just curious as to whether this is common while taking allopurinol to be having multiple attacks?”*
Decision to start urate-lowering therapy	*“Diagnosed gout last Sep, 3 flareups since. Didn’t take any meds so far, trying to be a father and worrying about impacts…... Should I take allporunol?”*
Medications for acute flares in real-time	*“I have a prescription for chochicine that I have never taken. I’m waiting on the dr office to call me back but I’m wondering if anyone would start taking it at this point in the attack?”*

### Questions about diet

There were 44 (12.3%) questions about dietary management. Sub-categories were questions about general dietary strategies, effectiveness of dietary changes as a management strategy, choice of alcoholic beverage, and the best weight loss strategies for people with gout. Examples of questions are shown in Table 4. There were also questions for specific advice about the value of many different foods, including yellow cheese, tart cherry, coffee, ostrich or emu meat, potato chips, rice, clams, and kombucha (fermented seaweed drink).

**Table 4:** Questions about diet

Sub-categories	Examples of questions
General dietary management	*“So I’ve finally decided to change my diet entirely…..I was just curious as to what my fellow gout sufferers eat on a daily basis so I can model a good diet for me to follow. What does everyone’s diet look like?”*
Effectiveness of dietary management	*“Has anyone been able to reach a consistent UA level of < 5 for that period of time just with diet change alone? If so, any suggestions?”*
Alcoholic beverage choices	*“My question for the people that STILL DO DRINK, do you have any regiments or rituals to live by? …….Do you strictly drink vodka and tart cherry juice cocktails??”*
Weight loss strategies	*“I am still trying my hardest to lose weight but I need to completely reorganize my diet so I can have a calorie deficiency but also enough nutrients. Any suggestions would help a lot.”* *“I was considering going back to a Ketogenic diet, and was considering intermittent fasting. Does anyone have any experience with IF and Keto for Gout?”*
Advice about a specific dietary factor	*“I was wondering what people’s experiences were with beans and coffee.”*

### Questions about non-pharmacological therapies

There were 37 (10.3%) questions about non-pharmacological management, including questions about the benefits of supplements, the role of activity during flares, choice of footwear, and topical treatments during a flare. Less frequent questions related to exercise strategies, devices and mobility aids during walking, and the value of blood donation or sex to improve gout.

There were 13 questions that specifically related to supplements. These supplements included curcumin, theracurmin, multivitamin, black cherry, vitamin C, “enzyme” supplements, herb gel, and alkaline water. Questions about several supplement combinations marketed for urate or gout were also posted including: ‘Gouch’ (includes cherry extract, ginger, quercetin, boerhavia, and couch grass), celery seed extract, stinging nettle root, ‘Urcinol’ (includes banana leaf, acai fruit extract, bicarbonate of soda, yucca root, turmeric, milk thistle, artichoke leaf, and celery seed), and ‘Uric Acid Support’ (includes vitamin B complex, vitamin C, tart cherry, quercetin, and vitamin E, turmeric, bromelain, and grape seed extract).

### Non-specific questions about gout management

There were 37 (10.3%) non-specific questions regarding gout management posted. These questions often included a detailed narrative about the course of the person’s experience, with a question requesting general advice from the community (for example, a detailed narrative of >500 words about the person’s previous experience, followed by “*Really open to suggestions... for anything, really…… I also know so little about gout, so any information is good information, tbh.”).* Requests for non-specific advice were also made during the initial flare of disease (“*First attack, too late in the evening to go to the doctor. What can I do to relieve symptoms right now?”)*

### Less frequent questions

A number of other categories were less frequently observed, with <10% questions per category (Table 1). These categories included questions about triggers, biological causes of gout, and serum urate testing. Questions about serum urate testing included the role of portable monitors for serum urate testing at home and interpretation of an individual’s serum urate results. There were some requests for social support (“*I’m 30 years old, and pretty upset that I may have to give up two of my favorite hobbies... Can someone help talk me down a bit*?”), and questions about financial and employment issues such as negotiating health insurance systems, disclosure of the condition to an employer, and the ability to work once a diagnosis of gout has been made. There were also questions about sourcing of expensive medications (“*They gave me a script for colchicine and i don't have insurance. ….. Any idea on how to get more in the future for cheaper?*”). Questions about reliable information primarily related to electronic sources or apps that can be used to help manage gout.

## Discussion

This analysis of questions posted on the social media website Reddit shows uncertainty about symptoms and management strategies for gout are common. The questions show that medications, particularly urate-lowering therapy, are often viewed negatively, with concern about side effects and the balance between risks and benefits. In this online community sample, there was a strong interest in non-drug strategies, including many different dietary solutions and supplements.

The most common single category was uncertainty about symptoms, particularly for attacks that did not conform to typical patterns. A recent detailed symptoms analysis of patients with crystal proven gout has shown that many patients do not present with stereotypical attacks, and that flares may affect more than just ‘classical’ joints such as the 1st metatarsophalangeal joints [13]. It is also likely that some community users were justifiably concerned about an incorrect diagnosis of gout. These data suggest that patients require a more detailed description of the range of gout symptoms from healthcare providers, including advice that prolonged symptoms are unusual and should prompt a request for further medical review.

Management categories were also common, emphasizing the information needs for patients for general management advice, medication advice, and non-pharmacological advice. The community users demonstrated concerns about long-term medications, and it appeared that the balance of benefits and potential side effects for some users was uncertain. The concern about flares during initiation of urate-lowering therapy highlights the need for further discussion with patients about the long-term benefits of ULT and efforts to reduce flares during initiation using anti-inflammatory prophylaxis [3].

There was a strong focus on non-drug management, including questions about the role of many dietary factors and supplements, including many with no reported benefits for gout. These findings are consistent with the lay view of gout as being caused by overindulgence in rich food and alcohol [14]. It is also consistent with our previous work describing media depictions of gout as a condition that should be primarily managed by dietary interventions [15].

Notable from the analysis of the postings was that there were very few questions concerning serum urate testing, results or targets. Given that serum urate lowering is the central strategy for long-term effective management of gout, these data suggest that information about this fundamental aspect of gout management has not widely penetrated the gout patient community. Together, these findings highlight the importance of changing prevailing social narratives of gout from a self-inflicted condition that can be primarily managed through dietary modification, to contemporary concepts of gout as a chronic disease of urate crystal deposition that can be effectively managed through complementary pharmacological and non-pharmacological urate-lowering strategies [3].

Study limitations include the lack of community user information. Reddit community users are known only by their usernames, and demographic and disease details are not available. Therefore, it is not possible to determine whether the questions are consistent with those of all people with gout, or indeed whether all the community users truly have the disease. Furthermore, disease duration was not available for all community users with gout, and we are unable to systematically determine whether the community user had a new diagnosis of gout, or had longstanding disease. It is possible that the information needs of Reddit community users do not represent all people with gout [16]. This study did not analyse social media sites other than Reddit; this site was chosen due to its large number of community users and dominant market share. Contributors to smaller online communities or other platforms may generate other categories. The study design included analysis of questions about gout, but not the responses to these questions by other community users; this analysis will form the basis of future work examining beliefs about disease management. The key study strengths are the large number of community users and the ability of this study to capture the questions of many individuals. A further advantage of the study design is the ability to capture questions about gout in real-time, often posted at the time of symptoms. This process reduces the risk of recall bias, which can be particularly problematic in studies of a flaring condition.

## Conclusions

This analysis of questions posted on Reddit highlights a number of information needs for people with gout. The key findings are uncertainty about symptoms and questions about disease management, both pharmacological and non-pharmacological. These findings may inform development of strategies to address the information needs of people with gout, with the ultimate goal of improving patient and community understanding about the disease and its management.
